# William Eglinton

**Published:** 1888-02

**Authors:** 


### HALL'S JOURNAL OF HEALTH

TRUTH DEMANDS NO SACRIFICE; ERROR CAN MAKE NONE.

VOL. 35. FEBRUARY, 1888. No 2.

### WILLIAM EGLINTON.

This world-renowned sensitive, was born in London, July 10, 1857. In early youth, although dreamy and imaginative to a remarkable degree, he gave no indication of those wonderful faculties which subsequently developed themselves. At the age of sixteen, his mother and almost only friend and counsellor, was called to another life. From this time, William, or Willie, as he was familiarly called, was left pretty much to his own impulses. His father was what is usually termed, a free-thinker, and a frequent attendant upon the meetings presided over by Mr. Charles Bradlaugh, a pronounced materialist, and, naturally enough, young Eglinton was fast drifting into a corresponding line of thought, when, in February, 1874, a discussion upon spiritualism took place at the hall where Mr. Bradlaugh was accustomed to speak, between two rival speakers, the one advocating and the other decrying the spiritual theory. This discussion young Eglinton attended, and remarked the disfavor with which the advocacy of the spiritual theory was received by the assemblage, but it having been there recommended that those who were willing to be convinced of its truth should seek for evidence in their own homes, the father of Willie and one of his neighbors, determined to try it, and, having provided themselves with a book of rules, commenced operations that very evening.

Willie, however, refused to join this circle, declaring, with the positiveness of ignorance, that it was "all humbug."

For seven or eight sittings no results were obtained, which afforded Willie an opportunity for ridicule, of which he freely availed himself, until, choosing between the penalty of making one of the circle or leaving the house during the sittings, he joined the company of the "lunatics," as he called them, "for the fun of the thing." Writing of these events he says: "a strange, mysterious feeling came over me which I could not shake off. I sat down at the table determined that if anything happened I would put a stop to it. Something did happen, but I was powerless to prevent it. The table began to show signs of life and vigor; it suddenly rose off the ground and steadily raised itself in the air until we had to stand to reach it. This was in full gaslight. It afterwards answered intelligently, questions which were put to it, and gave a number of test communications to persons present. The next evening saw us eagerly sitting for further manifestations, and with a larger circle, for the news had got widely spread that we had "seen ghosts and talked to them," together with similar reports. After we had read the customary prayer I seemed to be no longer of this earth. A most ecstatic feeling came over me and I presently passed into a trance. All my friends were novices in the matter, and tried various means to restore me, but without result. At the end of half an hour I returned to consciousness, feeling a strong desire to relapse into the former condition. We had communications which proved conclusively to my mind that the spirit of my mother had really returned to us. * * * I then began to realize how mistaken, how utterly empty and unspiritual, had been my past life, and I felt a pleasure indescribable, in knowing beyond a doubt, that those who had passed from earth could return and prove the immortality of the soul. In the quietness of our family circle, only broken by the admission of friends to witness the marvellous manifestations, we enjoyed to the full extent, our communion with the departed; and many are the happy hours I have spent in this way."

It was from this beginning that other circles were formed, other mediums developed and many a hard-headed materialist brought to a knowledge of the continuance of life in the world to come.

Here permit us to remark that there is scarcely a family of half a dozen members, which, by the exercise of the same degree of perseverance, would not be able to develop, at least, one of its number, as an instrument of intelligent communication between the two worlds. All that is required is that the members should approach the subject in a spirit of honesty, and, having assumed the proper attitude, earnestly desire and patiently await the coming of the mysterious visitors. The conditions are so simple as to be within the reach of every household. All that is required is a common deal table of moderate size and chairs for the sitters ; these may consist of any number, although our experience has been that two persons of opposite temperaments are better than more, owing, it may be presumed, to the difficulty of harmonizing so many different elements always more or less conflicting. It is not essential that the room should be dark nor that the sitters should touch hands, as was formerly advised. Indeed, we have obtained the best results in a subdued light, with the hands disconnected, resting lightly upon the table. If under these conditions the table be made to tip, treat it soberly and address yourselves to the invisible intelligence who has responded to your invitation, in a polite and becoming manner, avoiding all levity. Be patient, be decorous, for you must realize that you are in the presence of those who have passed on to higher planes of existence.

In conversing, ask questions which admit of a negative or affirmative reply only. Since the days of the Rochester rappings three tips or raps are understood to mean "yes," one, "no," and two of doubtful or uncertain import. Should your question call for a more difficult answer, as for example, a fact or a name, you may arrive at it by calling the alphabet and requesting that the right letter be denoted by a single tip, and so on to the end of the word or sentence. It is a tedious process, but the best that can be done with the means at hand. Numbers may be given in the same way. It is literally the a, b, c, of spirit intercourse, but many a sorrowing heart has been made glad, and many a soul gathered to a knowledge of the truth by this simple process.

From these sittings the development of young Eglinton's mediumship was rapid and satisfactory, including nearly all the phases with which investigators have become familiar, and in the fall of 1875 he began to sit for the public, and fairly entered upon the by no means envious career of a professional medium. The Secretary of the Experimental Committee of the British National Association of Spiritualists, in speaking of their experiments with Mr. Eglington, says : "Those who have attended the series of *seances* have reason to thank Mr. Eglinton for his thorough sincerity, simplicity and cordiality. * * * The orderly manner in which the *seances* have been conducted, the strict, yet simple tests imposed, and the facilities given to strangers to satisfy themselves of the *bona fide* nature of the manifestations, have had lasting and beneficial results "

In 1876, Mr. Eglinton held three series of twelve sittings each, at the instance of the members of the British Association, which extended over a period of more than nine months, in the course of which, he was subjected to the severest tests which the ingenuity of the sitters could devise, notwithstanding which, the manifestations which occurred were more marvellous than any which had preceded them, including materialization.

In summarizing the outcome of this series of *seances*, Mr. Farmer, in his late admirable biography of Mr. Eglington, says: "The methods were sound, and the results do not rest upon a single experiment. The facts were in many instances repeated, and results obtained at one *seance* verified over and over again at subsequent meetings."

Mr. Eglinton paid occasional visits to the Provinces, in compliance with the solicitation of those who were desirous of witnessing the phenomena presented through his instrumentality. About this time he was forced by illness to suspend for a period of some months all *seance* holding, later on Mr. Eglinton visited South Africa, and subsequently to that, at the invitation of some eminent scientific and literary Swedes, he was induced to visit them in their own country, where under favorable conditions he gave 19 consecutive *seances* at the houses of the *elite* in Stockholm, to the entire satisfaction and conviction of those who were fortunate enough to be present.

The report made of those *seances* by Professors Törnebom and Edland, after alluding to the testimony of such eminent and pains-taking investigators as Professors Wallace, Crookes, and Zöllner, and asserting the true character of the various manifestations witnessed by them in common with the other members of the circle, closes with these remarks, which we commend to investigators in general. "When something strange and inexplicable occurs, the first thing to do is not to find out if it is possible or not, but to get proofs that it really has been done. If we have tangible facts to put forward everybody must admit the possibility of the thing although they cannot explain it. Only those deny the reality of spirit phenomena who have never examined them, but pro found study alone *can* explain them. We do not know where we may be led by the discovery of these, as it seems, trivial occurrences, or to what new spheres of nature's kingdom they may open the way; but that they will bring forward important results, is already made clear to us by the revelations of natural history in all ages."

Taking leave of Stockholm Mr. Eglinton was induced to visit some of the leading cities of the Continent, by the earnest solicitation of eminent personages of both sexes. Wherever he went he was received with great cordiality and acquitted himself without a single drawback.

He subsequently paid a more extended visit to the Coutinent, and gave among others a series of twenty-five *seances* to Professor Zöllner and others of the University at Leipsic. Writing of Zöllner, Mr. Eglinton says, "I fell in love with him at once, he was so genial and open hearted. We had a continuous series of marvelous successes, Zöllner had intended writing a volume of his experiments, many of them novel, but death intervened."

Mr. Eglinton met with equal success in Vienna, where he was entertained by those high in authority. Writing of these *seances* Baron Hellenbach says, "I am not justified in calling that an impossibility, simply because it is utterly beyond my conception. In so doing I should be guilty of the same error as our scientists."

In the winter of 1881, Mr. Eglinton visited the United States. His initial *seances* were given in Boston and vicinity some accounts of which appeared in the *Banner of Light* of April 30, 1881. In Rhode Island among other manifestations, full form materializations occurred, which were fully recognized.

In the middle of April Mr. Eglinton came to the city of New York, upon the invitation of our well-known but rather skeptically inclined, fellow townsman, Charles D. Lakey Esq. at whose house he gave several *seances* which we were privileged to attend, the results of which were highly satisfactory. The manifestations included the levitation of the medium in two instances, to the high ceiling of the parlor; the floating overhead and from room to room of two large music boxes, which played or ceased to play at our command; the floating above the table of an etherealized, self-illuminated head, arms and bust of a figure, bearded and turbaned as in the far east; the independent writing and drawing upon blank white cards whilst shut within a book under the hand of some one of the circle; the materialization of full forms which were recognized, as well as other marvelous occurrences common to his mediumship.

Since his visit to this country Mr. Eglinton has spent a considerable time in India—that famous land of fakirs and occultism. Here it was that the most wonderful evidences of his medial powers were had, as marvelous indeed as any of which we have any account. It was here too that the famous prestidigitator "Keller" after testing Mr. Eglinton's mediumship publically acknowledged that it was altogether beyond his art and void of trick or deception. His more recent foreign trip was to Russia, where he was nobly entertained and commendably acquitted himself.

And here let us say that after a most careful examination into the facts of spirit phenomena, as they occur in the presence of that class of sensitives usually denominated mediums, running through a period of twenty-five years, we are able to affirm that we know it to be true in all its wonderful phases, as well as we know any one thing, the knowledge whereof, is acquired by the use of our senses.

Upon a subject of this importance, our readers have the right to demand of us an absolutely true statement. The genuineness of the manifestations occurring through Mr. Eglinton's instrumentality, having been questioned by a correspondent in the June (1886) number of the journal of the Society for Psychical Research (London). Mr. Eglinton responded to the charge in a flood of testimonials covering nearly forty pages of October *Light*, the writers being, for a large part, distinguished personages of both sexes, who, with few exceptions, gave the details of their several experiences, which, established beyond question, the independent character of the manifestations which occurred in their presence. There are no less than one hundred and seventy-three of these testimonials of persons distinguished in the various walks of life, including clergymen and men and women of marked scientific and literary attainments, whose statements are unquestioned by all who know them. We should fail in our duty if we neglected to lay before our readers these extraordinary facts which point unerringly, to the open door through which all shall enter upon an advanced state of existence in the world of spirits.

